# Transient postprandial increase in intact circulating fibroblast growth factor-21 levels after Roux-en-Y gastric bypass: a randomized controlled clinical trial

**DOI:** 10.7717/peerj.11174

**Published:** 2021-04-14

**Authors:** Mette S. Nielsen, Susanna Søberg, Julie B. Schmidt, Anne Chenchar, Anders Sjödin, Matthew P. Gillum

**Affiliations:** 1 Department of Nutrition, Exercise and Sports, Faculty of Science, University of Copenhagen, Frederiksberg, Denmark; 2 Novo Nordisk Foundation Center for Basic Metabolic Research, Faculty of Health and Medical Sciences, University of Copenhagen, Copenhagen, Denmark; 3 The Center of Inflammation and Metabolism and the Center for Physical Activity Research, Rigshospitalet, University of Copenhagen, Copenhagen, Denmark

**Keywords:** Fibroblast growth factor-21, Bariatric surgery, Randomized controlled trial, Diet-induced weight loss, Weight loss

## Abstract

**Background:**

Despite a consistent link between obesity and increased circulating levels of fibroblast growth factor-21 (FGF21), the effect of weight-loss interventions on FGF21 is not clear. We aimed to determine the short- and long-term effects of Roux-en-Y gastric bypass (RYGB) on intact plasma FGF21 levels and to test the hypothesis that RYGB, but not diet-induced weight loss, increases fasting and postprandial responses of FGF21.

**Method:**

Twenty-eight participants with obesity followed a low-calorie diet for 11 weeks. The 28 participants were randomized to undergo RYGB surgery at week 8 (RYGB group, *n* = 14), or to a control group scheduled for surgery at week 12 (*n* = 14). Fasting levels of intact, biologically active FGF21 (amino acids 1-181) and its postprandial responses to a mixed meal were assessed at week 7 and 11, and 78 weeks (18 months) after RYGB.

**Results:**

At week 11 (3 weeks after RYGB), postprandial responses of intact FGF21 were enhanced in participants undergoing surgery at week 8 (change from week 7 to 11: *P* = 0.02), whereas no change was found in non-operated control participants in similar negative energy balance (change from week 7 to 11: *P* = 0.81). However, no between-group difference was found (*P* = 0.27 for the group-week-time interaction). Fasting, as well as postprandial responses in intact FGF21, were unchanged 18 months after RYGB when both the RYGB and control group were collapsed together (change from week 7 to 78 weeks after RYGB: *P* = 0.17).

**Conclusion:**

Postprandial intact FGF21 levels were enhanced acutely after RYGB whereas no signs of sustained changes were found 18 months after surgery. When comparing the acute effect of RYGB with controls in similar negative energy balance, we failed to detect any significant differences between groups, probably due to the small sample size and large inter-individual variations, especially in response to surgery.

## Introduction

Fibroblast growth factor-21 (FGF21) is a recently discovered metabolic regulator with effects on energy regulation and glucose and lipid metabolism (Woo et al., 2013; Fisher & Maratos-Flier, 2016). FGF21 is also implicated in food preferences, with increased circulating FGF21 leading to a suppressed intake of sweets (Talukdar et al., 2016; Søberg et al., 2017). Paradoxically, increased circulating FGF21 levels are found in obesity, suggesting the potential existence of an FGF21 resistant state (Dushay et al., 2010; Fisher & Maratos-Flier, 2016).

Despite the clear link between obesity and FGF21, there is no consensus regarding the effect of weight-loss interventions on circulating FGF21 levels. Roux-en-Y gastric bypass (RYGB) can lead to impressive and sustained weight loss and induce major metabolic improvements with an acute resolution of type 2 diabetes and reduced cardiovascular risk (Schauer et al., 2003; Jørgensen et al., 2012; Aminian et al., 2019). Studies suggest both increased FGF21 levels 2–3 weeks and 3–6 months after RYGB (Jansen et al., 2011; Lips et al., 2014; Vienberg et al., 2017; Crujeiras et al., 2017) and unchanged levels up to 12 months after surgery (Woelnerhanssen et al., 2011; Ter Horst et al., 2017; Gómez-Ambrosi et al., 2017; Fjeldborg et al., 2017). When compared to similar weight loss induced by gastric banding, RYGB caused a marked increase in FGF21 concentrations in response to a mixed meal (Harris et al., 2017). If RYGB has a unique effect on FGF21 leading to increased circulating levels, it can be speculated that FGF21 might be involved in the metabolic improvements seen after RYGB (Patton, Khan & Kohli, 2017).

It is not clear whether a potential effect of RYGB on FGF21 is a weight-loss mediated effect, or if anatomical and physiological alterations caused by the surgical procedure have weight-loss independent effects on FGF21. Studies comparing the effect of RYGB and diet-induced weight loss show opposite effects (Lips et al., 2014; Gómez-Ambrosi et al., 2017; Crujeiras et al., 2017), with increased or unchanged FGF21 levels after surgery and decreased levels after diet-induced weight-loss interventions. However, so far, no studies have investigated this using a randomized controlled design including a control group in comparable negative energy balance in order to examine any potential direct effect of surgery. Furthermore, this is the first study to investigate changes in intact, i.e., bioactive, FGF21 after RYGB surgery.

We aimed to investigate short- (3 weeks) and long-term (18 months) changes after RYGB in fasting intact FGF21 and postprandial responses to a mixed meal. We, furthermore, tested the hypothesis that RYGB, but not diet-induced weight loss, had an acute effect on fasting and postprandial responses of intact FGF21, using a randomized controlled design including control patients on a low-calorie diet identical to that of RYGB treated patients.

## Materials and Methods

### Participants and study design

The study population, design, and methods have been described previously (Schmidt et al., 2016). Briefly, 28 obese, white Caucasians, aged 18–65 years, with a body mass index (BMI) ≥40 kg/m^2^ or BMI ≥35 kg/m^2^ in combination with obstructive sleep apnea or hypertension approved for RYGB, were consecutively recruited. Exclusion criteria included diabetes mellitus, thyroid dysfunction, hypothalamic or known genetic etiology of obesity, a current cancer diagnosis, use of drugs affecting energy metabolism, pregnancy, presence of contraindications to a low-calorie diet, substance abuse or smoking, and high intake of alcohol (men >14 drinks/week, women >7 drinks/week) or caffeine (>300 mg/day). Exclusion criteria were related to the main aim of the study (potential changes in energy expenditure after RYGB) which has previously been published (Schmidt et al., 2016).

The study was carried out from November 2009 to April 2013 and was approved by the Scientific Ethics Committees of the Capital Region of Denmark (journal no. H-2-2009-091) and was registered at clinicaltrials.gov (ID no NCT00939679). All study participants gave written informed consent.

The study included a visit in week 0, and three follow-up visits in week 7 and 11, and 78 weeks after RYGB (i.e., 18 months postoperatively). All visits were carried out at the Department of Nutrition, Exercise and Sports, University of Copenhagen. The participants were randomized to undergo RYGB surgery at week 8 (RYGB group), or to a control group scheduled for surgery at week 12. To ensure similar negative energy balance in both groups at the visits in week 7 and 11, the participants followed the same low-calorie diet throughout the first 11 weeks of the study period. Thus, the group operated in week 8, continued with the low-calorie diet 3 weeks after surgery. Self-evaluated compliance to the diet was registered at weekly meetings with a dietician.

#### Screening visit

Eligible patients were identified by one of two endocrinologists at Hvidovre Hospital. If inclusion criteria were fulfilled, the patient was scheduled for a screening visit where detailed information about participation was given.

#### Run in visit (week 0)

Anthropometric data and a fasting (12 h) blood sample were collected and the low-calorie diet was initiated. Randomization was carried out.

#### Follow-up visits (week 7 and 11, and 78 weeks after RYGB)

At 8:00 AM, a fasting (12 h) blood sample and anthropometric data were collected. Participants consumed a test meal within 20–30 min at 9:00 AM and postprandial blood samples were collected at 15, 30, 60, 90, 120, and 180 min after initiating the meal.

#### Low-calorie diet and test meal

The low-calorie diet consisted of 4 powder-meals (Cambridge Weight Plan^©^), milk, low-fat yogurt, and vegetables and provided 1,030 kcal/day (48 E% carbohydrates, 39 E% protein, and 13 E% fat). Both groups followed this diet for 11 weeks, except for the RYGB group on the day of surgery (500 ml non-caloric liquids) and postoperative days 1 and 2 (powder-meals providing 410 kcal/day). The test meal consisted of the same powder-meal provided throughout the low-calorie diet period (310 kcal, 42 E% carbohydrates, 41 E% protein, and 17 E% fat, 175 ml).

#### Biochemical measures

Blood samples were analyzed for FGF21 and bile acids (bile acids only at week 7 and 11). Blood was drawn into chilled EDTA tubes for FGF21 and chilled dry tubes for bile acids. Samples in dry tubes were left to coagulate for 30 min before centrifugation, whereas the remaining samples were immediately cooled on ice and centrifuged at 4 °C. All blood samples were frozen at −80 °C until analyzed. Samples were analyzed for concentrations of intact FGF21 taking into account the recently described proteolytic cleavage of FGF21 by dipeptidyl peptidase IV and fibroblast activation protein (Zhen et al., 2016; Dunshee et al., 2016). FGF21 was analyzed using N- and C-terminally directed antibodies to detect full-length, active protein according to the manufacturer’s instructions (F2131; EagleBiosciences, Nashua, NH, USA). Total bile acids were analyzed using an enzyme cycling based TBA assay kit (cat. no. DZ042A-K; Diazyme, San Diego, CA, USA).

### Statistical analysis

The sample size calculation was based on the primary endpoint (24-h energy expenditure) of the study (Schmidt et al., 2016).

Descriptive data summaries are reported as mean ± standard deviation (SD). Differences in baseline characteristics between RYGB and controls were assessed using a two-sample *t*-test.

Changes in fasting levels of FGF21 from week 7 to 11 in the RYGB group and the control group and changes from week 7 to 78 in both groups together were analyzed using a linear mixed model with week as a fixed effect. Changes compared to the control group were analyzed using a linear mixed model with a group-week interaction. Changes in fasting levels from week 0 to 7 are presented as a pooled analysis with both groups together and were analyzed with a linear mixed model with week as a fixed effect. All models were adjusted for age and gender and included a subject-specific random effect.

Changes in postprandial responses of FGF21 from week 7 to 11 in the RYGB group and the control group and changes from week 7 to 78 in both groups together were analyzed using a repeated measurement linear mixed model with a week-time interaction. Changes compared to the control group were analyzed using a repeated measurement linear mixed model with a group-week-time interaction. In case a significant week-time interaction or group-week-time interaction was found, pairwise comparisons for the change from baseline to each time point during the meal test were carried out using post hoc, model-based approximate *t*-tests. All models were adjusted for age and gender and included a within-visit subject-specific random effect. Non-normally distributed data were log-transformed.

Furthermore, associations between changes in the area under the curve (AUC) for FGF21 and changes in AUC for bile acids were evaluated using simple linear regressions. AUC was calculated by the trapezoidal rule.

Results are shown as mean and 95% confidence interval (CI). Graphs with postprandial responses are based on raw data and presented as mean ± SEM. *P*-values less than 0.05 were considered significant. Statistical analyses were conducted in R version 3.3.2 (R Development Core Team, 2020) and figures were produced using GraphPad Prism version 8.0.1.

## Results

**Figure 1:**
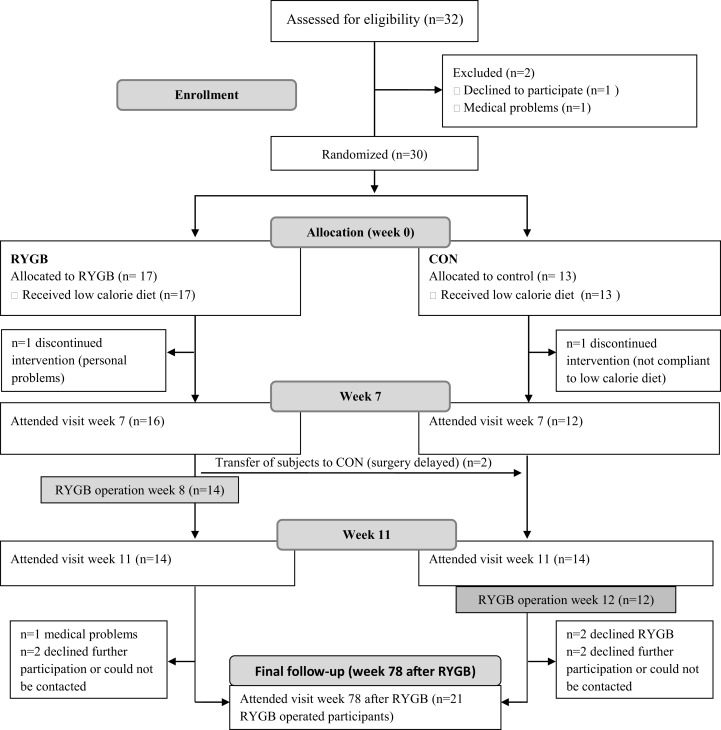
Study flow. Thirty-two participants attended the screening visit, but two participants dropped out before the visit in week 0. One subject from each group dropped out after randomization, but before the visit in week 7. Due to delay in surgery, two participants were transferred from the RYGB group to the CON group. To ensure an equal distribution of participants in the two groups, two additional participants were allocated to the RYGB group, thus resulting in 14 participants in each group. From week 11 to the last visit 78 weeks after RYGB, seven subjects were lost to follow-up. RYGB, Roux-en-Y gastric bypass, CON, control.

Twenty-eight participants completed the run-in visit (week 0) and the follow-up visits in week 7 and 11. Fourteen participants were randomized to RYGB surgery in week 8, and 14 participants to receive surgery in week 12 and thus served as a control group. Twenty-one participants completed the follow-up visit 78 weeks (18 months) after RYGB, and one participant had no blood samples from the follow-up visit (Fig. 1). Baseline characteristics are shown in Table 1.

**Table 1:** Baseline characteristics.

	All (*n* = 28)	RYGB grp (*n* = 14)	Control grp (*n* = 14)
Age (years)	39.6 ± 10.1	37.7 ± 10.4	41.6 ± 9.8
Gender (females)	20 (71%)	10 (71%)	10 (71%)
Weight (kg)	135.3 ± 19.6	133.4 ± 19.7	137.3 ± 20.1
BMI (kg/m^2^)	45.8 ± 4.4	45.4 ± 5.1	46.2 ± 3.9
Fat mass (kg)	67.3 ± 12.1	67.1 ± 14.3	67.6 ± 10.0
Fat free mass (kg)	66.4 ± 11.1	64.9 ± 8.1	68.0 ± 13.6

**Note:**

Data are shown as mean ± standard deviation or proportions (%). BMI: body-mass index.

From week 0 to week 7, BMI decreased by 4.4 [4.0, 4.9] kg/m^2^ in a pooled analysis of both groups together (*P* < 0.01). From week 7 to 11, BMI loss in the RYGB group was 2.6 [2.1, 3.1] kg/m^2^ (*P* < 0.01). This decrease in BMI was greater compared to the BMI loss in the control group (control: 1.7 [1.2, 2.2] kg/m^2^, *P* < 0.01). From week 7 to 78 weeks after RYGB, BMI decreased with 10.9 [9.2, 12.7] kg/m^2^ (*P* < 0.01) for the 20 participants with valid data from the final visit (pooled analysis of both groups together).

### Effect of 7 weeks of a low-calorie diet on fasting FGF21

We were not able to detect an effect of the preoperative diet-induced weight-loss period (from week 0 to 7) on fasting intact FGF21 levels in a pooled analysis of both groups together (week 0: 154.7 [124.7, 184.8] pg/ml, week 7: 138.9 [108.9, 168.9] pg/ml, *P* = 0.26) (Table 2A).

**Table 2:** Changes in fasting intact plasma FGF21 from (A) week 0–7 (both groups together), (B) week 7–11 in the RYGB group, (C) week 7–11 in the control group, and (D) week 7–78 (both groups together).

	*n*	Week 0	Week 7	Week 11	Week 78 (after RYGB)	Change
(A) RYGB + CON	28	154.7 [124.7, 184.8]	138.9 [108.9, 168.9]			−15.9 [−44.9, 13.2]
(B) RYGB	14		153.0 [90.6, 215.5]	164.1 [101.7, 226.5]		11.0 [−55.8, 77.8]
(C) CON	14		126.0 [77.6, 174.5]	127.5 [79.0, 175.9]		1.4 [−23.0, 25.8]
(D) RYGB + CON	20		142.0 [100.1, 183.8]		118.1 [76.1, 160.2]	−23.8 [−63.8, 16.1]

**Note:**

Data are shown as mean and 95% CI. All *P* > 0.05 when analyzed using a linear mixed model with week as a fixed effect. RYGB: Roux-en-Y gastric bypass; CON: control.

### Short- and long-term effects of RYGB on fasting and postprandial FGF21

When analyzing data in the RYGB group operated in week 8, we were not able to detect any changes in fasting intact FGF21 3 weeks after RYGB (week 7: 153.0 [90.6, 215.5] pg/ml, week 11: 164.1 [101.7, 226.5] pg/ml, *P* = 0.71). Furthermore, no significant changes in fasting intact FGF21 was found 78 weeks (18 months) after RYGB for the 20 participants with valid data from the final visit (week 7: 142.0 [100.1, 183.8] pg/ml, week 78 after RYGB: 118.1 [76.1, 160.2] pg/ml, *P* = 0.21) (Tables 2B and 2D).

**Figure 2:**
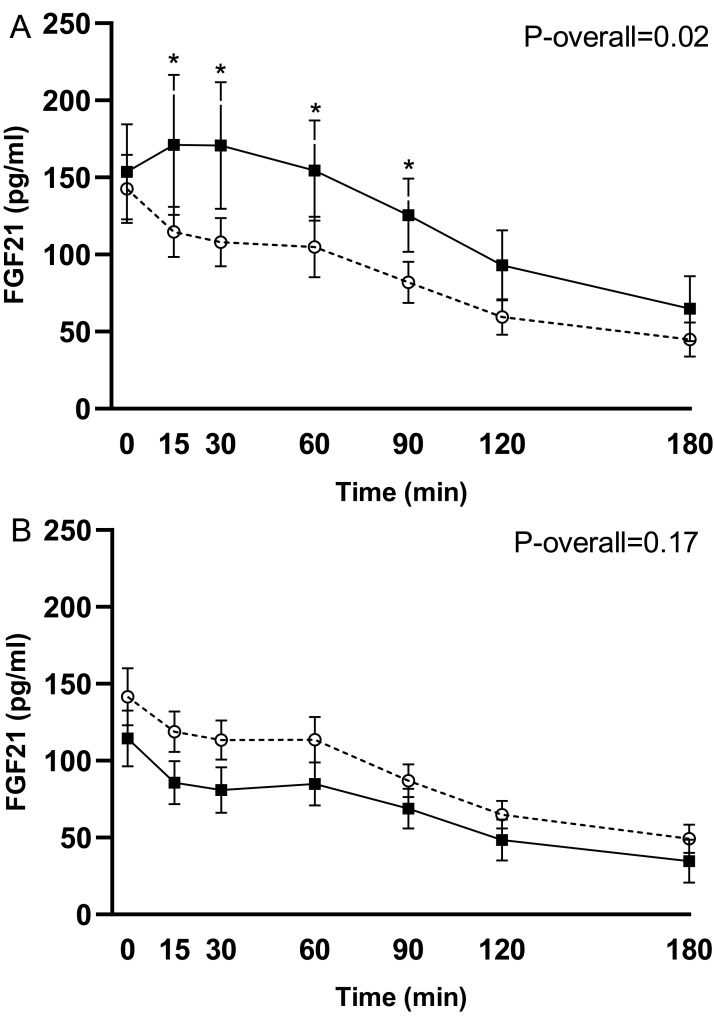
Plasma levels of intact FGF21 in response to the test meal after 7 weeks on a low-calorie diet (○, dotted line) and 3 weeks postoperatively (◼, filled line) in the 14 subjects undergoing RYGB in week 8 (A). Plasma levels of intact FGF21 after 7 weeks on a low-calorie diet (○, dotted line) and 18 months postoperatively (week 78 after RYGB) (◼, filled line) in the 20 subjects with valid data at the last follow-up visit (B). Data are shown as mean ± standard error of the mean. *P*-overall was obtained from a repeated measurement linear mixed model including a visit-time interaction and age and gender as fixed effects and patient as a random effect. Time points with differences between visits were identified through model-based pairwise comparisons. *P < 0.05.

In response to the test meal, postprandial concentrations of intact FGF21 increased 3 weeks after RYGB in the group operated in week 8 (change from week 7 to 11: *P* = 0.02 for the time-week interaction). This increase occurred mainly in the early postprandial period (time 0–90 min) (*P* ≤ 0.03 for pairwise comparisons) (Fig. 2A). The postprandial FGF21 response was, however, unchanged 78 weeks (18 months) after RYGB in the pooled analysis for the 20 participants with valid data (change from week 7 to 78: *P* = 0.17 for the time-week interaction) (Fig. 2B).

### Comparison of RYGB and diet-induced weight loss on fasting and postprandial FGF21 (the period from week 7 to week 11)

No changes in fasting intact FGF21 was found in the control group from week 7 to week 11 (week 7: 126.0 [77.6, 174.5] pg/ml, week 11: 127.5 [79.0, 175.9] pg/ml, *P* = 0.90) (Table 2C), and we were not able to detect any difference in fasting intact FGF21 between participants operated in week 8 and participants operated in week 12 (control group) when comparing changes from week 7 to week 11 (*P* = 0.76 for the week-group interaction).

**Figure 3:**
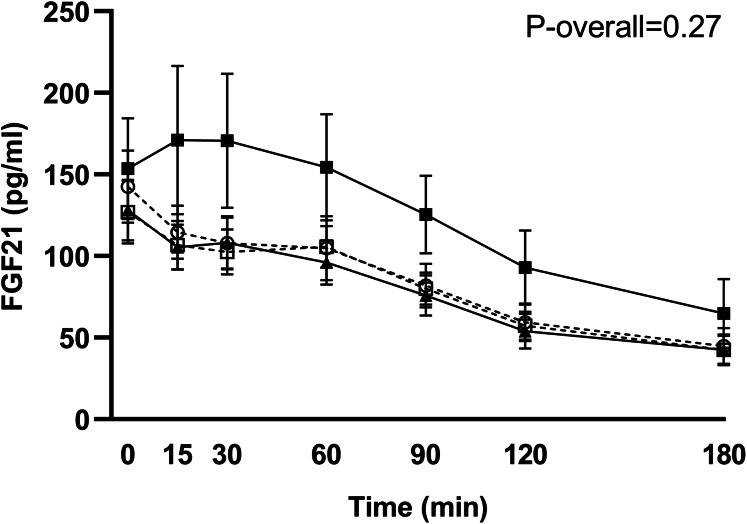
Plasma levels of intact FGF21 in response to the test meal in subjects scheduled for RYGB after 7 weeks on a low-calorie diet (○, dotted line), in control subjects after 7 weeks on a low-calorie diet (□, dotted line), in RYGB subjects after 11 weeks on a low-calorie diet + RYGB surgery (◼, filled line) and in control subjects after 11 weeks on a low-calorie diet (▴, filled line) . Data are shown as mean ± standard error of the mean. *P*-overall was obtained from a repeated measurement linear mixed model including a group-visit-time interaction and age and gender as fixed effects and patient as a random effect.

Postprandial concentrations of intact FGF21 were unchanged in the control group (change from week 7 to 11: *P* = 0.81 for the time-week interaction), and no significant differences in FGF21 responses from week 7 to 11 between the RYGB group and the control group were found (*P* = 0.27 for the group-time-week interaction) (Fig. 3).

### Associations between FGF21 and bile acids 3 weeks after RYGB

Changes in AUC for total bile acids were positively associated with changes in AUC for FGF21 3 weeks after surgery in the RYGB group (*P* = 0.02, *R*^2^ = 0.55).

## Discussion

Intact FGF21 levels in response to a mixed meal increased during the early postprandial phase acutely after RYGB but returned to pre-surgical levels 18 months after surgery. When comparing acute changes after RYGB to that of a control group in similar negative energy balance, we were not able to show differences in fasting or postprandial responses between RYGB treated patients and controls. However, our data do show large interindividual variations in FGF21, especially in response to surgery. No significant changes in fasting intact FGF21 were seen after the preoperative diet-induced weight-loss period or 3 weeks or 18 months after RYGB.

Our results indicate an acute effect of RYGB on postprandial FGF21 responses. This effect could potentially be caused by the anatomical and physiological alterations after surgery directly affecting FGF21. More rapid delivery and absorption of ingested nutrients, particularly glucose, to the liver after RYGB may increase hepatic FGF21 production (Harris et al., 2017). Plasma FGF21 increases acutely by oral glucose ingestion possibly through activation of the carbohydrate responsive-element binding protein (Iizuka, Takeda & Horikawa, 2009; Lin et al., 2012). Furthermore, increases in bile acids after RYGB may affect FGF21 secretion through hepatic activation of the bile acid receptor farnesoid-X receptor (FXR), which has been identified as a signaling pathway for FGF21 gene expression and secretion (Cyphert et al., 2012). Increases in bile acid concentrations in the early postprandial period (time 0–60 min) have been reported 3 weeks after RYGB in the same cohort as analyzed in the present study (Schmidt et al., 2016). In a posthoc analysis, changes in AUC for total bile acids were positively associated with increases in AUC for FGF21 3 weeks after surgery.

Although, we were not able to detect any significant differences in FGF21 responses between RYGB-patients and control patients in the same negative energy balance, the graphical presentation of data (Fig. 3) could indicate opposite effects of RYGB and diet-induced weight loss. This is also seen when calculating percentages change in AUC from week 7 to 11. The FGF21 response increased by 42% in the RYGB group and was unchanged in the control group (decrease of 0.1%). The low number of participants in each group (*n* = 14) combined with the large interindividual variations in both fasting and postprandial responses of FGF21, especially after surgery, could explain the lack of significance between groups. Fasting levels at week 7 varied from 23 to 324 pg/ml in the 28 included participants. Large variations have previously been found in healthy normal-weight subjects, with fasting levels ranging from 21 to 5,300 pg/ml (Gälman et al., 2008). Previous studies comparing the effect of bariatric surgery and diet-induced weight loss all show opposite effects (Lips et al., 2014; Gómez-Ambrosi et al., 2017; Crujeiras et al., 2017). Lips et al. (2014) compared the effect of RYGB and a very-low-calorie diet (VLCD) and found elevated levels of fasting total FGF21 and responses to a mixed meal 3 weeks after RYGB whereas calorie restriction lowered FGF21 levels (Lips et al., 2014). Although not supported by our data, it is possible that RYGB can counteract weight-loss induced effects on FGF21 and that the effect of surgery is a weight-loss independent effect.

Postprandial responses of intact FGF21 were unchanged 18 months after surgery, indicating that FGF21 only transiently increases after RYGB. Previous studies tend to support these findings with enhanced FGF21 levels in the early postoperative period (Jansen et al., 2011; Lips et al., 2014; Vienberg et al., 2017; Crujeiras et al., 2017) and unchanged levels 12 months after surgery (Ter Horst et al., 2017; Gómez-Ambrosi et al., 2017; Fjeldborg et al., 2017). Whether the increase in FGF21 acutely after RYGB contributes to the metabolic improvements after surgery needs further investigation.

The longitudinal design assessing both short- and long-term effects of RYGB on FGF21 is a strength of this study. The pair-fed design furthermore makes it possible to compare postoperative changes in FGF21 with a control group consuming the same standardized diet. However, despite our attempt to ensure similar energy intake in both groups, a limitation of this study was the somewhat larger weight loss in the RYGB group compared to the control group. The low number of participants is, furthermore, likely to limit the possibility to detect between-group differences due to the high heterogeneity in FGF21 responses following RYGB surgery. Furthermore, the lack of a control group matched for weight loss 18 months after surgery does not allow us to evaluate if RYGB may attenuate a decrease in FGF21 caused by the substantial weight loss. Lastly, a mixed-meal test is not the most potent inducer of FGF21 secretion (Vienberg et al., 2017). Thus, it is possible that we would have detected a larger increase in FGF21 responses after a carbohydrate-rich, instead of a mixed-meal challenge.

## Conclusions

In conclusion, postprandial responses in FGF21 enhanced acutely after RYGB but were unchanged 18 months after surgery. Although we were not able to show significant differences in FGF21 responses between RYGB-operated and control participants in a similar negative energy balance, our data do show large interindividual variations, especially in response to surgery. Thus, it is possible that our sample was too small to detect such between-group differences and that RYGB surgery may well have an acute weight loss-independent effect on FGF21.

## Supplemental Information

10.7717/peerj.11174/supp-1 Supplemental Information 1 CONSORT checklist.

10.7717/peerj.11174/supp-2 Supplemental Information 2 Raw data. Categorical data are described in the head of each column containing such data.

10.7717/peerj.11174/supp-3 Supplemental Information 3 Protocol. Register based research protocol in Danish.

## Additional Information and Declarations

### Data Availability

The following information was supplied regarding data availability: Raw data are available in the Supplemental Files.

### Clinical Trial Registration

The following information was supplied regarding Clinical Trial registration: The study was approved by the Scientific Ethics Committees of the Capital Region of Denmark (journal no. H-2-2009-091) and was registered at clinicaltrials.gov (ID no NCT00939679).
