# Supplementary material for: Transient postprandial increase in intact circulating fibroblast growth factor-21 levels after Roux-en-Y gastric bypass: a randomized controlled clinical trial
Source: PeerJ. 2021 Apr 14;9:e11174. doi: 10.7717/peerj.11174 (PMC8052976; doi:10.7717/peerj.11174)
Supplement: Supplemental Information 3 — Register based research protocol in Danish. [file peerj-09-11174-s003.pdf]

Protokol for Register Substudie til:

(Register-Studie baseret på biologisk materiale fra biobank)

Titel på hovedstudierne: GO Bypass (H-3-2013-138) og ERGEM studiet (H-2-2009-091)

Titel på Registerprotokol: Effekten af en fedmeoperation på FGF21

Forsøgsansvarlig:

Professor Anders Sjödin,
Institut for Idræt og Ernæring,
Københavns Universitet,
Rolighedsvej 26, 1958 Frederiksberg C
Danmark
amsj@nexs.ku.dk

---

1) Baggrund og problemstilling

Hormonet Fibroplast growth factor 21 (FGF21) menes at have en rolle i energi- og glukosemetabolismen, og der er for nyligt også kommet fokus på hormonets rolle ift. appetitreguleringen og indtag af søde fødevarer (Søberg et al. 2017). Det er derfor muligt, at FGF21 kan være en ny spiller i behandlingen af fedme, ved at kunne påvirke appetitten samt reducere indtag af kalorieholdige søde fødevarer.

Få studier har undersøgt effekten af en fedmeoperation på FGF21. Disse studier tyder på, at der er en effekt at operationen på niveauer af FGF21 (Lydia-Ann et al. 2017, Lips et al. 2014), men det er ikke klart, om effekten skyldes operationen i sig selv, eller om det er en effekt af det postoperative vægttab. Med vores kohorter (ERGEM og GO Bypass studierne), vil det være muligt for os at undersøge effekten af operationen på FGF-21 niveauer sammenholdt med en kontrolgruppe i tilsvarende negative energibalance (ERGEM data). Det vil med dette data, derfor være muligt at belyse, om effekten af operationen skyldes anatomiske ændringer koblet til selve operationen, eller om det blot er en effekt af det store vægttab der følger med en fedmeoperation. Desuden vil vi med GO Bypass kohorten blandt andet kunne undersøge hvordan mulige ændringer i FGF-21 hormonet er koblet til appetitreguleringen, indtag af søde fødevarer samt ændringer i smagen sød. Dette vil kunne bidrage til ny viden omkring hormonet, og dets mulige betydning for behandling af fedme.

2) *Formålet med dette studie er derfor at afklare følgende problemstillinger:*

Formålet med dette studie er dels at undersøge effekten af Roux-en-Y gastric bypass (RYGB) på FGF21 niveauer, og hvorledes disse ændringer adskiller sig fra en kontrolgruppe i tilsvarende negative energibalance (data fra ERGEM studiet)

Ligeledes ønsker vi at undersøge ændringer i FGF21 efter RYGB og Sleeve gastrectomy (SG) samt, hvorledes disse ændringer er associeret til ændringer i

præferencer for smagen sød, samt indtag af søde fødevarer (data fra GO Bypass studiet).

Undersøgelse af ændringer i FGF21 efter RYGB er beslægtet med formålene i hovedstudierne (GO Bypass og ERGEM).

3) Hypotese

Vores hypotese er, at RYGB vil fører til øgede niveauer af FGF21 sammenlignet med en kontrolgruppe som endnu ikke har undergået RYGB operation, men som har tabt et tilsvarende antal kg på en lav kalorie diæt (data fra ERGEM studiet).

Vores hypotese er ligeledes, at RYGB og SG vil fører til øgede niveauer af FGF21 og at disse vil være associeret til ændringer i præferencer for sød smag, samt indtag af søde fødevarer (data fra GO Bypass studiet).

4) Forsøgsmetode og design

EDTA-plasma i blodprøverne analyseres for niveauer af FGF-21 efter gældende standarder. Disse analyseres i faste prøver og i 6 prøver taget i intervallet 15-180 min efter et standardiseret måltid (postprandielle prøver).

I ERGEM studiet er der taget faste prøver ved baseline, samt faste og postprandielle prøver efter 7 ugers lav-kalorie diæt, og 11 ugers lav-kaloriediæt (+operation i operationsgruppen). Dette studiedesign bruges ligeledes til at undersøge effekten af en fedmeoperation (operationsgruppen) mod en kontrolgruppe på tilsvarende lavkalorie diæt, men som ikke er blevet opereret (kontrolgruppe).

I GO Bypass studiet er der taget faste og postprandielle prøver cirka 3 måneder og 2 uger inden en fedmeoperation, samt 6 og 18 måneder efter operationen. Vi ønsker her at undersøge effekten af operationen på FGF-21 niveauer samt at sammenholde disse niveauer med andre målinger foretaget på GO Bypass studiet. Disse målinger inkludere: antropologiske målinger, smagspræferencer og smagssensitivitet, præferencemålinger fra et buffet måltid, neuralaktivitet i hjernens belønningscentre samt appetitregulerende hormoner og mål for den metaboliske profil.
I kraft af godkendelsen fra Datatilsynet til GO Bypass har vi hjemmel til at have data på projektet og videregivelse til dette register forskningsprojekt da det er inden for samme formål at data analyseres og behandles.

5) Statistiske overvejelser

Power er beregnet ud fra det primære endpoint på henholdsvis ERGEM (energiforbrug) og GO Bypass studiet (vægttab). Da kun få studier har undersøgt effekten af en fedmeoperation på niveauer af FGF-21, er denne undersøgelse fortsat eksplorativ, hvilket vanskeliggør muligheden for at beregne power baseret på FGF-21.

6) Forsøgspersonerne

Inklusions og eksklusionskriterier knyttet til GO Bypass studiet er listet nedenfor. Data fra i alt 43 forsøgspersoner vil blive analyseret fra GO Bypass studiet.

Inklusionskriterierne er følgende: patienter der er henvist til Bariatrisk Klinik på Køge Sygehus med henblik på RYGB eller Sleeve operation.

Eksklusionskriterierne er følgende: graviditet samt manglende evne, fysisk eller mentalt, til at overholde de procedurer der kræves for overholdelse af forsøgsprotokollen, dette vurderes af forsøgspersonalet. Derudover er følgende eksklusionskriterier forbundet med MR-scanningen: Tidligere eller nuværende neurologisk sygdom, pacemaker eller andet implanteret elektronisk apparatur, indopererede metalgenstande, svær klaustrofobi. Desuden gælder, at der kun inkluderes forsøgsdeltagere, hvis omfang (fysiske størrelse) muliggør scanning. Dette sikres ved informationssamtalen. Inklusion til MR scanning er ikke forudsætning for deltagelse i de andre dele af studiet.

Inklusions og eksklusionskriterier knyttet til ERGEM studiet er listet nedenfor. Data fra i alt 28 forsøgspersoner vil blive analyseret fra ERGEM studiet. I henhold til metode beskrevet i ERGEM protokollen, blev 14 randomiseret til kontrolgruppen og 14 til operationsgruppen.

Inklusionskriterierne er følgende:
· Alder 18-65 år
· BMI ≥40, eller ≥35 i kombination med obstruktiv søvnapnø eller forhøjet blodtryk
· Planlagt til RYGB operation på Hvidovre Hospital
· Skal have tabt mellem 1-4% af kropsvægten efter 3 måneders kostvejledning

Eksklusionskriterierne er følgende:
· Vurderet til ikke at være kvalificeret til RYGB operation af lægerne på Hvidovre Hospital
· Diabetes Mellitus
· Ikke Kaukasere
· Kropsvægt >160kg (pga. begrænsninger med DEXA-scanneren), eller morfologiske forhindringer for at kunne være i DEXA-scanneren (>40cm i maksimum liggende anterior-posterior dimension, eller >60cm i maksimum liggende kropsbredde)
· Hæmoglobin <7.0 mmol/L
· Patienter på specifikke diæter (f.eks. vegetarer, Atkins)
· Tidligere tilfælde af thyroidea dysfunktion, eller anvendelse af thyroidea medicin
· Hypothalamus-relateret eller genetisk ætiologi af fedme
· En aktuel cancerdiagnose
· Klaustrofobi
· Tidligere sygdomstilfælde af diverticulitis
· Andre operationer end RYGB planlagt i de 3 måneder efter RYGB
· Stofmisbrug eller rygning
· Anvendelse af receptpligtig eller håndkøbsmedicin, der influerer på metabolismen, vægt, eller appetit
· Højt alkoholindtag (kvinder >14 genstande/uge, mænd >21 genstand/uge)
· Højt koffeinindtag (>300 mg/dag)
· Forekomst af faktorer, der taler imod anvendelse af en LCD, inklusiv:
o Tidligere forekomst af hjerte arytmi, og/eller brug af antiarytmiske medicin

o Nedsat nyrefunktion (creatinin clearance <60 mL/min)
o Leverenzymer (alanin aminotransferase (ALAT) eller aspartat aminotransferase (ASAT)) >3x øverste grænse af normalområdet
o Mælkeallergi eller laktose intolerans
o Porphyria eller phenylketonuria
o Tidligere sygdomstilfælde af urinsyregigt
o Amning
o Brug af monoamine oxidase inhibitorer eller ikke-kalium bevarende diuretika
o Manglende evne eller vilje til at følge LCD protokollen
· Ikke kan lide den pulverbaserede måltidserstatning, som anvendes i studiet
· Ikke kan lide yoghurt, mælk, eller gulerødder (da disse er essentielle elementer af den
anvendte Lavkaloriediæt)

Så det samlede antal FP er 71 i dette studie

Biobanken der er oprettet efter hovedstudierne er anmeldt til Datatilsynet ved SCIENCE' løbende godkendelse journalnummer 2015-57-0116.

Forskningsbiobanken er anmeldt til Datatilsynet ved SCIENCE' løbende godkendelse journalnummer 2015-57-0116.

Til dette studie skal anvendes 0,5 ml blod. Materialet opbevares i forskningsbiobanken højst til projektets afslutning, det vil sige når analyserne er udført og data er valideret og eventuelle reanalyser er udført, som formodes at være september 2018. hvorefter evt. rester destrueres.

Dispensationsansøgning:

Der blev i forbindelse med indhentelse af informeret samtykke til GO Bypass studiet indhentet skriftligt samtykke til oprettelse af en biobank. FP blev informeret om frivilligheden ved at donere til biobank samt at deres biologiske materiale udelukkende vil anvendes som forskningsbiobank i kommende beslægtede forskningsprojekter efter at disse har opnået godkendelse hos Det Videnskabsetiske Komitésystem. Der er derfor allerede indhentet informeret samtykke jf. hvilket deltagerne accepterer om oprettelse af biobank. Der søges derfor om dispensation for indhentelse af nyt samtykke jf. komitélovens § 10.
Det bekræftes hermed, at det biologiske materiale er udtaget, mens de pågældende personer var i live, og at materialet er indsamlet og opbevaret efter de gældende regler herfor (Persondataloven). Projektet vil ikke indebære sundhedsmæssige risici eller på anden måde være til belastning for forsøgspersonerne, da primær-studiet er afsluttet.
Analyserne kan ikke kobles til sygdomme eller andre sundhedsmæssige parametre hos FP og derfor vurderes det som helt usandsynligt, at analysen vil føre til fund af sundhedsmæssig betydning eller alvorlige, sygdomme, som det efterfølgende ville være relevant at oplyse forsøgspersonerne om. Såfremt der mod al forventning kan tolkes noget vil FP blive kontaktet såfremt de ikke har frabedt sig dette i samtykkeerklæringen.

Data inklusive biologisk materiale fra studiet ERGEM er fuldstændig anonymiseret, da godkendelsen fra Datatilsynet som gives via vores fællesanmeldelse på SCIENCE (2015-57-0117/2015-57-0116) til at have data som Personhenførbare udløb i 2017.

Vi har derfor anonymiseret data jf. Datatilsynets retningslinjer og dette er vurderet muligt, da vi har et antal FP på 28 (større end 5), data er ikke så komplekse at den enkelte forsøgsperson kan identificeres hverken ud fra enkelte data (sjældne sygdomme eller andet) eller ud fra en kombination af deres data. Anonymiseringen er fortaget ved at alle nøgler der kan dekode data er destrueret således at ingen, ej heller forsker, er i stand til at genetablere identiteten af deltagerne.

ERGEM deltagerne blev i deltagerinformationen til hovedstudiet informeret om, at det biologiske materiale i biobanken kun ville blive anvendt såfremt et kommende beslægtet studie var godkendt af VEK.
Vi har altså ikke oplyst dem om, at deres materiale ville blive destrueret.

Derfor vurderer vi, at vi kan anvende det anonymiserede biologiske materiale til forskning, uden at det er anmeldelsespligtigt, da det er lovligt opsamlet.

7) Videnskabetiske overvejelser

Studiet vil blive gennemført i overensstemmelse med de etiske principper i den gældende version af Helsinki Deklarationen (Fortaleza, Brazil, October 2013), Der er ingen sundhedsmæssig gevinst for de personer hvis biologiske materiale indgår i studiet, men det vurderes at det videnskabelige og sundhedsmæssige fordel af studiets resultater overstiger de ulemper det indebærer for personerne der frivilligt har doneret deres biologiske materiale til biobanken i forbindelse med deres deltagelse i hovedstudierne.

8) Økonomisk Støtte

Professor Anders Sjödin har taget initiativ til projektet og udgifterne til løn og materiale til udførsel af analyserne dækkes af GO Bypass projektet, der er en del af Governing Obesity initiativet, som har modtaget støtte fra Københavns Universitets 2016-fond (31,7 millioner d.kr.).

9) Forsøgspersonernes data

Alle oplysninger behandles fortroligt og personhenførbare data opbevares efter gældende lovgivning jf. Persondataloven. Forsøgspersonens identitet vil gennem hele forsøget være kodet med et forsøgspersonnummer og fødselsdato.
Ved offentliggørelse af forsøgets resultater vil forsøgspersonens identitet ikke blive afsløret.
Forsøget er dækket af Fakultetet SCINCE generelle udtalelse fra Datatilsynet til at håndtere personfølsomme oplysninger i videnskabelige forsøg (journalnummer: 2015-57-0117).

10) Publikation

Studiets resultater vil uanset om de er positive, negative eller inkonklusive blive offentliggjort i faglige internationale tidsskrifter og/eller på videnskabelige møder og NEXS hjemmeside Studiet er registeret på clinicaltrials.gov

11) Referencer

Lips MA, de Groot GH, Berends FJ et al. Calorie restriction and Roux-en-Y gastric bypass have opposing effects on circulating FGF21 in morbidly obese subjects. Clinical Endocrinology 2014; 81:862-870.

Lydia-Ann LS, Gordon SH, Smith GI et al. Roux-en-Y gastric bypass surgery has unique effects on postprandial FGF21 but nor FGF19 secretion. J Clin Endocrinol Metab, 2017; 10:3858-3864.

Søberg S, Sandholt CH, Jespersen NZ et al. FGF21 is a sugar-induced hormone associated with sweet intake and preference in humans. Cell Metabolism 2017; 25:1045-1053.
